# Combining SDAE Network with Improved DTW Algorithm for Similarity Measure of Ultra-Weak FBG Vibration Responses in Underground Structures

**DOI:** 10.3390/s20082179

**Published:** 2020-04-12

**Authors:** Sheng Li, Xiang Zuo, Zhengying Li, Honghai Wang, Lizhi Sun

**Affiliations:** 1 National Engineering Laboratory for Fiber Optic Sensing Technology, Wuhan University of Technology, Wuhan 430070, China; wanghh@whut.edu.cn; 2 School of Information Engineering, Wuhan University of Technology, Wuhan 430070, China; zuoxiang@whut.edu.cn (X.Z.); zhyli@whut.edu.cn (Z.L.); 3 Department of Civil and Environmental Engineering, University of California, Irvine, CA 92697-2175, USA; lsun@uci.edu

**Keywords:** similarity measure, subway tunnel, distributed vibration, feature extraction, autoencoder, ultra-weak FBG

## Abstract

Quantifying structural status and locating structural anomalies are critical to tracking and safeguarding the safety of long-distance underground structures. Given the dynamic and distributed monitoring capabilities of an ultra-weak fiber Bragg grating (FBG) array, this paper proposes a method combining the stacked denoising autoencoder (SDAE) network and the improved dynamic time wrapping (DTW) algorithm to quantify the similarity of vibration responses. To obtain the dimensionality reduction features that were conducive to distance measurement, the silhouette coefficient was adopted to evaluate the training efficacy of the SDAE network under different hyperparameter settings. To measure the distance based on the improved DTW algorithm, the one nearest neighbor (1-NN) classifier was utilized to search the best constraint bandwidth. Moreover, the study proposed that the performance of different distance metrics used to quantify similarity can be evaluated through the 1-NN classifier. Based on two one-dimensional time-series datasets from the University of California, Riverside (UCR) archives, the detailed implementation process for similarity measure was illustrated. In terms of feature extraction and distance measure of UCR datasets, the proposed integrated approach of similarity measure showed improved performance over other existing algorithms. Finally, the field-vibration responses of the track bed in the subway detected by the ultra-weak FBG array were collected to determine the similarity characteristics of structural vibration among different monitoring zones. The quantitative results indicated that the proposed method can effectively quantify and distinguish the vibration similarity related to the physical location of structures.

## 1. Introduction

Over the past decades, with the rapid development of rail transit infrastructure in China, the operation safety and security of subway systems have attracted much attention. According to the recent research progress of distributed optical fiber-sensing technology [1,2,3,4,5,6,7], the requirement for time- and space-continuous monitoring for the geotechnical underground structures [8] has gradually become feasible. Comparisons between various commonly used sensors for underground structure monitoring were reported in [9,10], which revealed that the ultra-weak fiber optic Bragg grating (FBG) array [11] can be used for both static and dynamic measurements [12,13,14]. In the field of dynamic measurement, it was reported that the distributed vibration detected by the ultra-weak FBG array can be applied to track train and identify incursion [10,15]. Moreover, the change of the structural vibration responses usually reflects the evolution of the structure state to a certain extent. A wide range of research reports concerning the vibration-based structural condition assessment can be found in [16,17,18,19]. Compared with ground transportation, the daily operation of underground trains is of obvious regularity. For example, the speed of trains in each travel zone always follows the operation schedule, and the number of passengers does not change suddenly within a certain period due to commuting habits. Moreover, the temperature and humidity fields of underground infrastructure are relatively stable due to the management measures of tunnel ventilation. Therefore, it can be assumed that the structural vibration responses corresponding to the excitation of multiple passing trains in a certain structural state should be stable and similar. With the support of distributed vibration monitoring adapted to the long-distance underground structures, it is possible to quantify the structural status by measuring the similarity of structural vibration responses for a specified monitoring area under different stages and this is the research motivation of the paper.

The vibration responses of subway tunnel structures can be regarded as a collection of typical one-dimensional time-series signals. The similarity measure between time series can often be converted to measure the distance between vectors. The Euclidean distance (ED) [20] and its variants based on common L_p_-norm [21] are the most straightforward methods for similarity measures of such one-dimensional time-series. However, there is a slight difference in the length of duration in the vibration responses excited by each train passing through the monitoring area, making the ED and its variants unable to directly perform the similarity measure for unequal-length sequences. Even when dealing with equal-length vibration signals, these methods are susceptible to noise and time misalignment and are unable to deal with local time-shifting. Dynamic time warping (DTW) [22] is an option to overcome time-shifting, which allows a time series to be either stretched or compressed to provide a better match with another time series. Therefore, it can be used to handle similarity measures between inconsistent length sequences. Another group of similarity measures suitable for processing unequal-length time series is developed based on the concept of the edit distance for strings [23]. Compared with DTW which only considers the constrain bandwidth, the similarity measure based on the edit distance requires tuning more parameters [24,25,26] to find the most similar set of matching patterns. It is reported [15] that the data amount is huge for vibration responses detected by ultra-weak FBG of each monitoring area under the excitation of passing trains. This often results in high time complexity and is expensive in terms of processing and storage costs to directly use the above methods to perform a similarity measure on the raw format of high-dimensional vibration responses of underground structures. Furthermore, it is difficult to completely avoid random outlier interference during data collection and transmission. Therefore, the results of the similarity measure based on any algorithm may significantly deviate from expectations if the raw signals are not carefully wrangled.

Feature extraction should be the most intuitive idea to solve the above problems. It can improve the effectiveness and efficiency of the similarity measure by maintaining the characteristics of the original signal in a smaller dimensionality. Compared with principal component analysis (PCA) [27], linear discriminant analysis (LDA) [28] and other linear feature extraction methods, manifold learning [29], restricted Boltzmann machine (RBM) [30], autoencoder (AE) [31], as typical representatives of non-linear feature extraction methods, can retain much richer sample features of high-dimensional vibration signals. High computational complexity is the bottleneck of manifold learning based on local domain classification and its feature extraction process is sensitive to noise [32]. Therefore, this method is not suitable for extracting the characteristics of the vibration responses of underground structures that cannot avoid noise interference. RBM and its derivative deep belief network [33] use the probability distribution rather than the real-valued sequence to express the characteristics of the hidden layer. These two methods for dimensionality reduction are not suitable for the similarity measure of real-valued sequences. The training of AE resembles that of the RBM. However, models of AE can be easier to train than that of RBM with contrastive divergence and are thus preferred in contexts where RBM training is less effective [34]. Adding a denoising process makes AE models substantially more robust to input variations or distortion, causing the deep network formed by a stacked denoising autoencoder (SDAE) with higher accuracy than that of the stacked autoencoder (SAE) [35,36]. Thus, the SDAE network is used to achieve feature extraction before the similarity measure in this paper. In the following second section, the implementation process of the proposed similarity measure is introduced in combination with typical one-dimensional datasets in the public UCR (University of California, Riverside) time-series data archives [37]. This part also illustrates the metrics used to evaluate the effectiveness of feature extraction and similarity measure. After that, based on the ultra-weak FBG vibration response of the actual underground structure, the feasibility and significance of the proposed similarity measure method in engineering are discussed.

## 2. Methodology and Implementation of Signal Similarity Measure

### 2.1. Overview of the Procedure for Signal Similarity Measure

**Figure 1:**
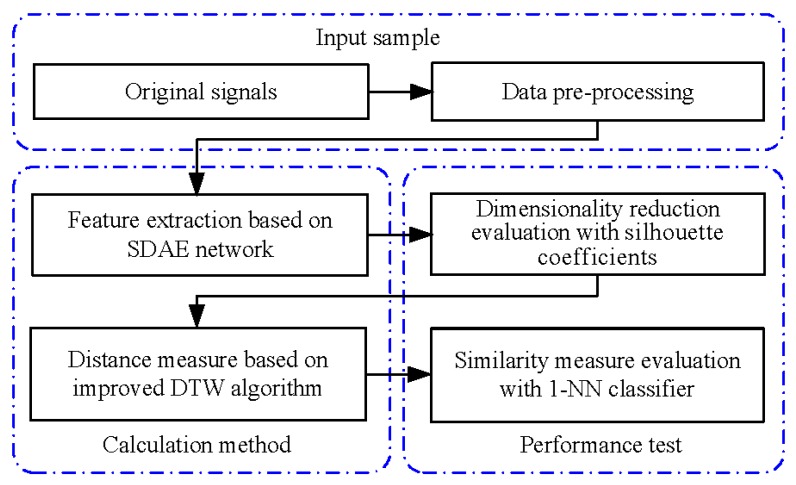
Similarity measurement and evaluation process.

As shown in Figure 1, the proposed method and performance test constituted the processing flow for the similarity measurement of one-dimensional time-series signals. The method in the left part of Figure 1 contained dimensionality reduction of the original sequence through feature extraction based on the SDAE network and distance measurement for the extracted feature sequences of equal length through the improved DTW algorithm. The silhouette coefficients and one nearest neighbor (1-NN) classifier in the right part of Figure 1 were used to evaluate the performances of feature extraction and distance measure, respectively.

**Figure 2:**
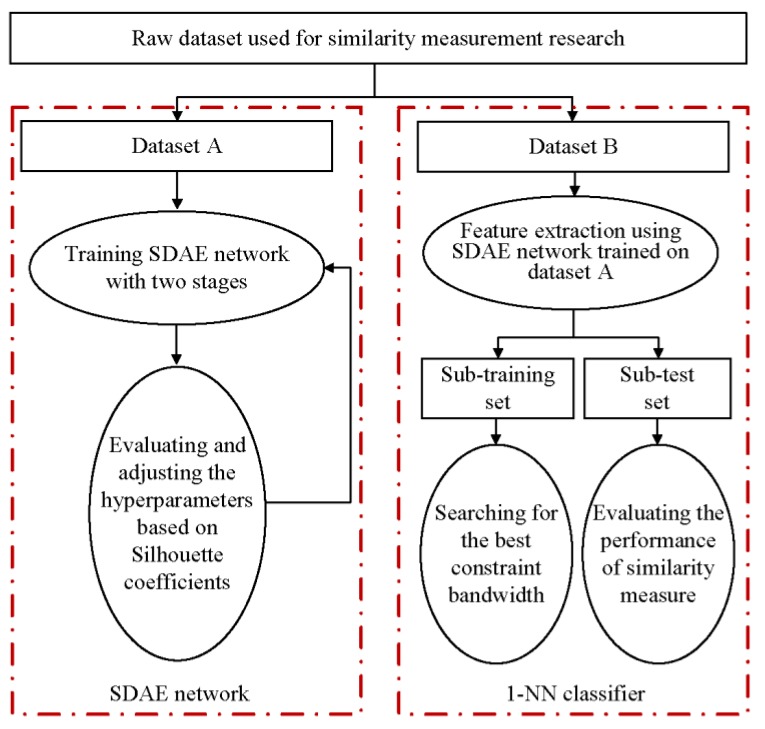
Functions of the dataset used for similarity measurement during implementation.

For the general time series involving the similarity measure, the need for dataset partitioning and the purpose of each divided dataset are shown in Figure 2, which primarily included two parts, namely, the unsupervised learning through the SDAE network and the supervised learning through the 1-NN classifier. Since the cost of collecting and processing the distributed high-dimensional vibration responses is often expensive or even prohibitive, two datasets with data labels (CinCECGTorso and SemgHandMovementCh2 [38]) were selected from the UCR time series archives to help explain the implementation process. The two selected datasets have moderate sample sizes and relatively long sequence lengths, ensuring the operation feasibility of dimensionality reduction based on the SDAE network under acceptable computing overhead. Moreover, both the selected datasets and the vibration of interest face some negatives in common, such as redundant information and outliers, which should be overcome when measuring the similarity of sequences, although their appearance and type are varied. The default training set and test set ratio of each dataset in UCR databases are different. For each of the selected raw datasets used for the subsequent research, we first merged the training and test sets, then shuffled the samples, and finally set a uniform split ratio to form datasets A and B. Table 1 shows the final processing results, in which the ratio of datasets A to B was three. Note that other partitioning ratios were also acceptable as long as the dataset to be partitioned had a sufficient sample size to ensure corresponding algorithm training. The Python packages Tensorflow and Keras, as well as their libraries [39], were utilized to establish the SDAE network and calculate different distance metrics, in which the operation of the 1-NN classifier that can choose different distance measurement methods referred to in the work by Regan [40].

### 2.2. Feature Extraction Based on Stacked Denoising Autoencoder (SDAE) Network

**Figure 3:**
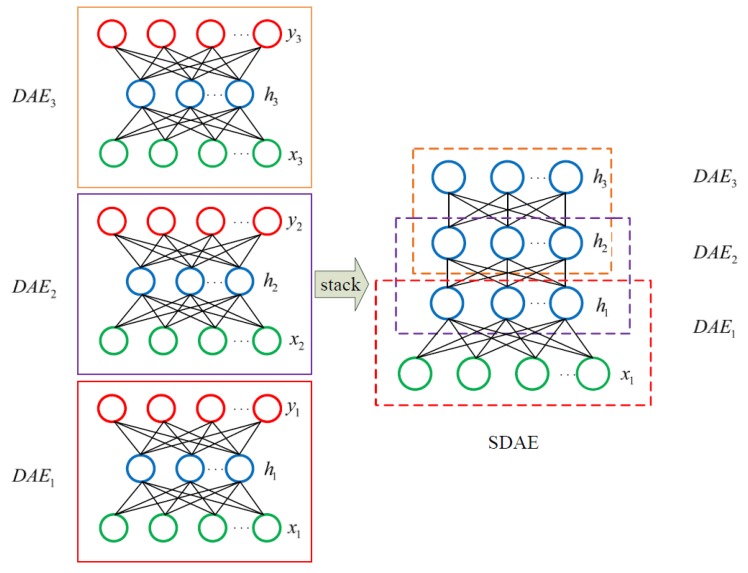
Schematic diagram of the stacked denoising autoencoder (SDAE) network stacking process.

As shown in the left part of Figure 2, feature extraction was primarily based on unsupervised training to shorten the sequence length in the second column of Table 1. Here, labels were just a supplement to fine-tune in the second training stage of the SDAE network, which was generally stacked by multiple three-layer DAE models. Figure 3 shows the structure of a typical SDAE network, which was formed by stacking three sub-DAE networks. Because the noise was actively added to the input data, hidden layers in such networks can retain more robust sample features during the learning process [41]. Here, greedy layer-wise training [42] that can boost the network learning efficiency was a preferred solution to conduct the pre-training process. In the first stage of feature extraction, the initial features of the input sample can be forcibly extracted through the unsupervised learning network. To obtain a better feature extraction effect, labels of the input sample were used to establish a classification output layer to perform a supervised training. Thereafter, a feature extraction model based on the SDAE network can be obtained through training the dataset A in Table 1. When a new sample dataset B was fed into the trained model, the feature representation of the last hidden layer can be regarded as reduced-dimensional features of the original input.

### 2.3. Dimensionality Reduction Evaluation with Silhouette Coefficients

**Figure 4:**
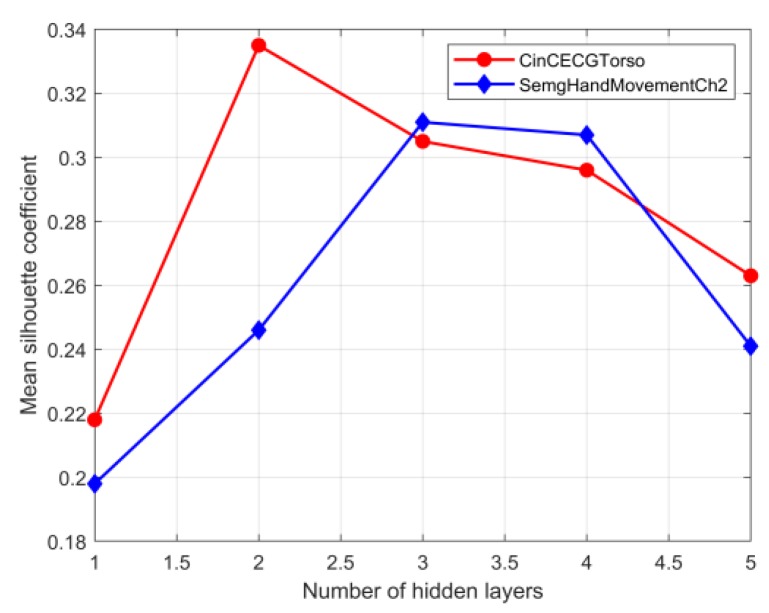
Mean silhouette coefficients at different number of hidden layers.

**Figure 5:**
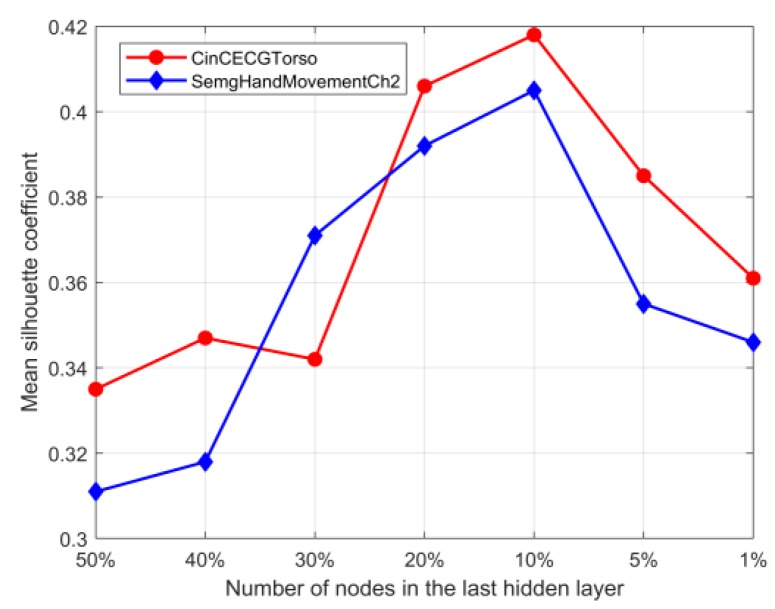
Mean silhouette coefficients at different reduction dimensionality.

**Figure 6:**
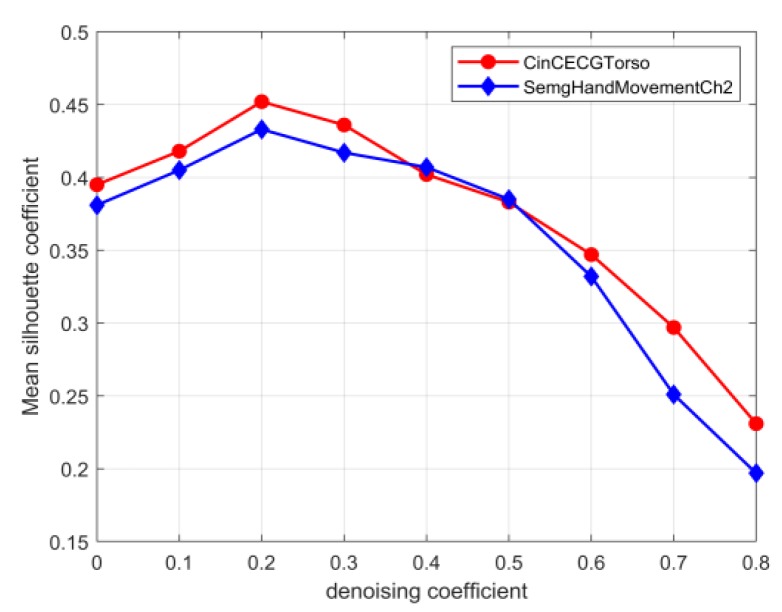
Mean silhouette coefficients at different denoising coefficients.

Through the above processing, a sequence having a length shorter than that of the original sequence listed in the second column in Table 1 can be obtained. It was straightforward that the effect and rationality of dimensionality reduction needed to be evaluated, indicating that the hyperparameter settings of the SDAE network should be assessed. Ideally, the feature vector generated due to dimensionality reduction should be able to represent the category information of the original sample to the greatest extent, namely, good feature extraction results should make the dimensionality reduction sequences belong to the same category closer, and the distance between the dimensionality reduction sequences belong to different categories farther. Silhouette coefficients [43] described as Equation (1) provide a single value measuring both the above two traits.

(1) $$s_{i}=\frac{b_{i}-a_{i}}{\max(a_{i},b_{i})}$$

where $s_{i}$ is the silhouette coefficient for observation i, $a_{i}$ is the mean distance between i and all observations of the same class, and $b_{i}$ is the mean distance between i and all observations from the different classes. Silhouette coefficients range between −1 and 1, with 1 indicating dense, well-separated different categories. Therefore, the mean silhouette coefficient for all observations can be used to evaluate the impact of the selection of various key hyperparameters on the performance of feature extraction based on the SDAE network for the two UCR datasets in Table 1. The operation based on grid search [44] combined with cross-validation [45] can guarantee to find the most accurate set of hyperparameter settings within a specified range, but it required iterating through all possible parameter combinations, which was very time-consuming in the face of large datasets and multiple parameters of interest. Another feasible option was to optimize the hyperparameter set step by step. Considering the characteristics of the SDAE network, the key hyperparameters that are usually concerned are the number of network layers, the number of hidden layer nodes, and the noise level [41]. As shown in Figure 4, the number of hidden layers of the SDAE network can be firstly determined by the mean silhouette coefficient. Here, in the training process, the adaptive moment estimation optimizer [46] was used to search the right learning rate automatically, and the maximum number of training epochs can be controlled based on the early stopping [47] technique. Other hyperparameters under the different number of hidden layers were derived through trial-and-error under the control of maximum number of training epochs and minimum reconstruction errors. As shown in Figure 4, when the number of hidden layers of the datasets CinCECGTorso and SemgHandMovementCh2 were set to two and three, respectively, the node number of the last hidden layer that reflected the dimensionality reduction effect can be further analyzed. Here, the hyperparameter configurations determined through trial and error in the previous step were used as the initial settings for the next tuning step and the key hyperparameter determined in the previous step remained constant in the subsequent tuning step. As shown in Figure 5, the number of nodes in the last hidden layer was expressed as a percentage of the original sequence length. After the number of hidden layers and the number of nodes in the last hidden layer were determined in turn, the reasonable value of the denoising coefficient [48] in the input layer of the SDAE network can be discussed. Figure 6 gave the relationship between different denoising coefficient and corresponding silhouette coefficient based on the tuning strategy of hyperparameters mentioned above. Hence, based on the hyperparameters determined by the maximum mean silhouette coefficients, the network structures of the SDAE for the two selected datasets used to obtain the dimensionality reduction sequences can be established.

**Figure 7:**
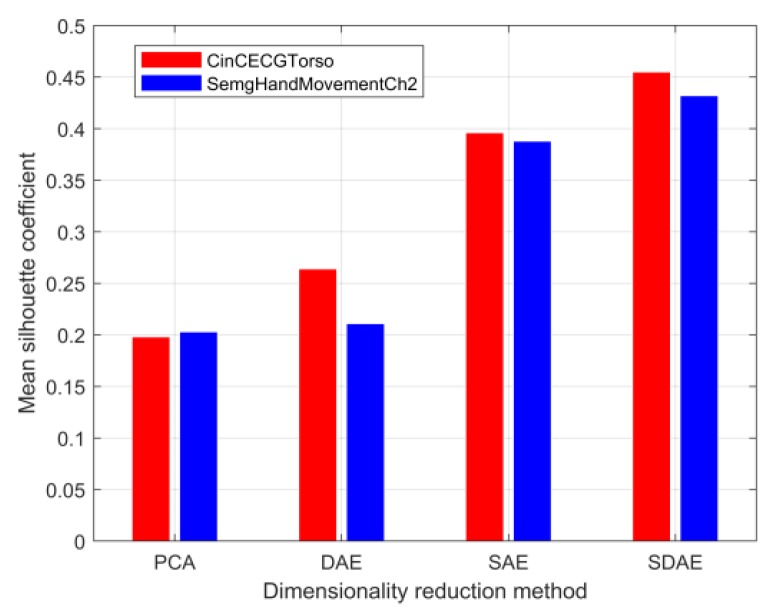
Comparison of different dimensionality reduction methods.

After the above step-by-step tuning of hyperparameters, the optimal mean silhouette coefficients of the datasets CinCECGTorso and SemgHandMovementCh2 with the feature extraction dimensions reduced to 20% of the original sequence length were 0.455 and 0.432, respectively. Figure 7 further shows that the capability of SDAE-based feature extraction was significantly better than that of other methods, although all the silhouette coefficient results did not exceed 0.5. Here, various comparison methods maintained a unified feature dimension reduction scale. Therefore, the SDAE network training process used for feature extraction for dataset B listed in Table 1 in this section was the premise for the subsequent similarity measure of reducing dimensionality sequences.

### 2.4. Distance Measure Based on Improved Dynamic Time-Warping (DTW) Algorithm

**Figure 8:**
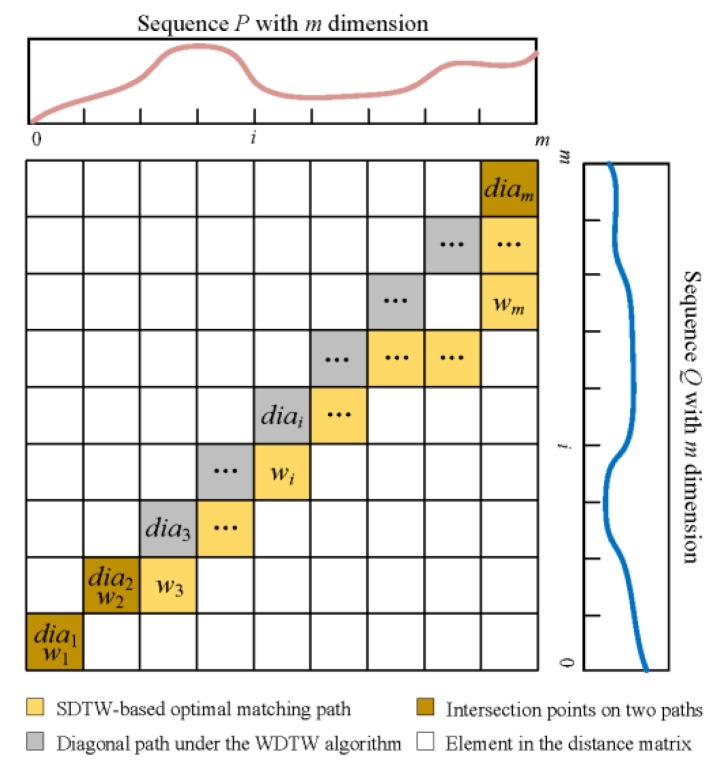
Warping offset distance expressed by the diagram of the DTW distance matrix.

Since the features were extracted as dimensionality reduction sequences of equal length, the impact of high time complexity and low calculation efficiency can be effectively avoided when measuring distance based on the DTW algorithm. Although the reported window-based constraint methods have some positive effects on avoiding the DTW’s matching path from falling into the suboptimum under certain circumstances, improvements against the influences of undesired warping [49] still deserve attention. Based on the DTW with a constraint of Sakoe–Chubaband [50] (hereinafter abbreviated as SDTW), warping offset distance ($d_{\mathrm{WOD}}$) was defined in the proposed improved DTW algorithm to further mitigate the effects of undesired warping. The defined $d_{\mathrm{WOD}}$ was the area between the optimal matching path and the diagonal path under the SDTW algorithm. As shown in Figure 8, these two paths were derived from the distance matrix D of two equal-length sequences after feature extraction, and the $d_{\mathrm{WOD}}$ described in Equation (2) can be shown as the cumulative sum of the differences between each point on the optimal matching path and each corresponding point to the unbiased state. By aligning the feature points of two sequences processed by the SDAE network, this method not only ensured that the matching path can recognize the slight warping of the time axis but also realized the constraint on the length of the matching path. Detailed definition of the distance matrix of DTW and the searching method of the optimal matching sequence based on dynamic programming can be found in [51]:

(2) $$d_{\mathrm{WOD}}=\sum_{i=1}^{m}\left|w_{i}-dia(i)\right|$$

where $w_{i}$ and dia(i) represent the *i*-th point in the optimal matching path and the *i*-th point in the diagonal of the distance matrix D, respectively. The sum of $d_{\mathrm{WOD}}$ and the distance based on the SDTW ($d_{\mathrm{SDTW}}$) was used as the distance metric of the improved DTW algorithm in Equation (3) and therefore $d_{\mathrm{similarity}}$ was regarded as the result of the similarity measure:

(3) $$d_{\mathrm{similarity}}=d_{\mathrm{SDTW}}+d_{\mathrm{WOD}}$$

### 2.5. Similarity Measure Evaluation with One Nearest Neighbor (1-NN) Classifier

The bandwidth r defines the constraint range of the matching path in the distance matrix and suppresses the influence of undesired convergence in the matching path [52]. Because there was a correlation between the defined warping offset distance and the SDTW algorithm, as well as the SDTW-based distance and the constraint bandwidth r, different r not only affected the optimal matching path of the SDTW but also led to the change of $d_{\mathrm{similarity}}$. The r determined the efficacy of the proposed similarity measurement method. It was reported that the 1-NN classifier on labeled data was a feasible way to evaluate the efficacy of the selected distance metric and its classification accuracy directly reflected the effectiveness of the similarity measure [53]. Moreover, the 1-NN classifier can be used to search for a proper r and the idea was to train a labeled dataset with different bandwidth constraints based on two distance metrics $d_{\mathrm{SDTW}}$, $d_{\mathrm{WOD}}$, respectively. Then, two sets of classification error rates $E_{\mathrm{SDTW}}(r)$ and $E_{\mathrm{WOD}}(r)$ at different r through the 1-NN classifier model can be derived. We defined $E_{\mathrm{SUM}}$ as the sum of $E_{\mathrm{SDTW}}(r)$ and $E_{\mathrm{WOD}}(r)$ and the constraint bandwidth r that minimized $E_{\mathrm{SUM}}$ was considered to be the appropriate choice for calculating $d_{\mathrm{similarity}}$.

**Figure 9:**
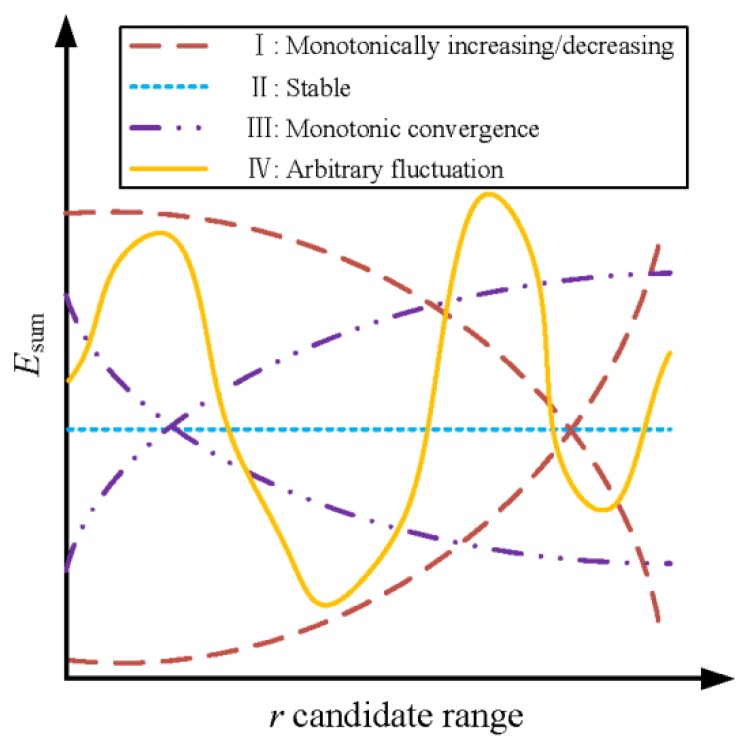
Typical variation of the sum of classification error rates at different constrains.

Figure 9 depicted the possibly typical variation of $E_{\mathrm{SUM}}$ at different r. For cases Ⅰ and Ⅳ, it was easy to determine the appropriate r based on the minimum $E_{\mathrm{SUM}}$. For case Ⅱ, it can be considered that the constraint bandwidth did not affect the distance measured by the SDTW algorithm, and the first r corresponding to the minimum can be seen as the candidate. For the situation in case Ⅲ that multiple candidate values within the convergence region corresponded to the same minimum value $E_{\mathrm{SUM}}$, the median of these candidate values was selected as r. Here, the general rules for determining and adjusting the preset range for r can refer to [52].

**Figure 10:**
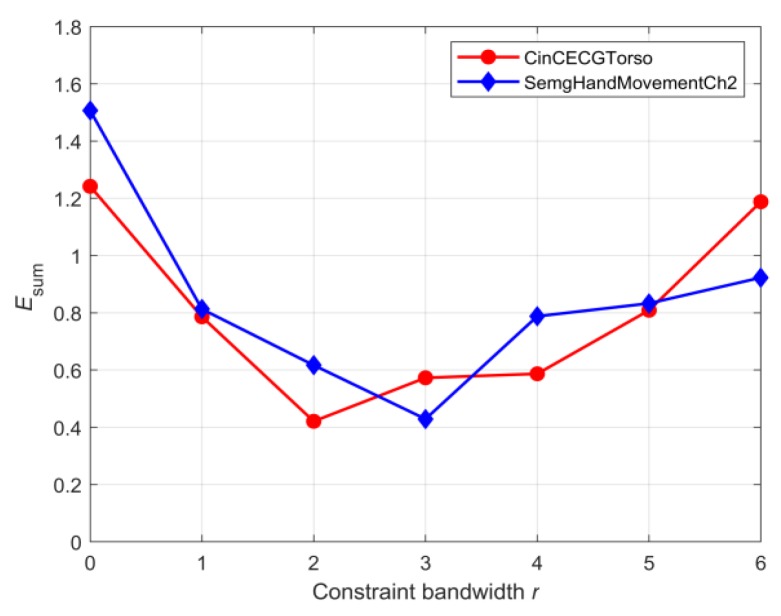
Variation of the defined error of two datasets with different constraint bandwidths.

**Figure 11:**
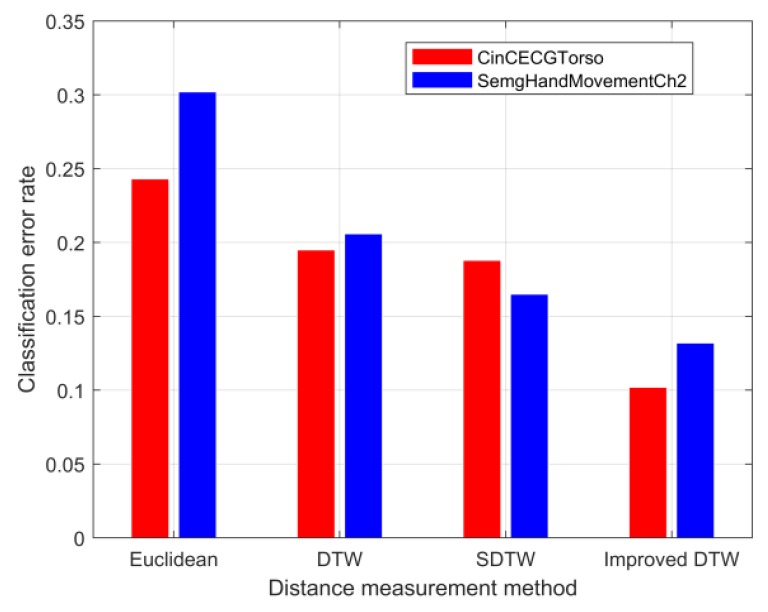
Performance comparison of 1-NN classifier under different distance metrics.

According to the data-processing procedure in the right part of Figure 2, the dataset B listed in Table 1 was further divided into sub-training and sub-test sets after the dimension reduction through the SDAE network. The dataset information used for the supervised learning of the 1-NN classifier was given in Table 2. Here, the sample size of the test set was made significantly larger than that of the training set according to the ratio commonly adopted in the dataset sheet of UCR archives [38]. First, the best r was searched based on the training results of the 1-NN classifier under two distance metrics. Figure 10 showed the variation of the classification error rate $E_{\mathrm{SUM}}$ of two datasets with respect to r after the dimensionality reduction in Table 2, and r for datasets of CinCECGTorso and SemgHandMovementCh2 should choose 2 and 3, respectively. Next, the defined $d_{\mathrm{similarity}}$ under the specified r was used as the distance metric of the 1-NN classifier to perform supervised training on the sub-training set. Also, other distance metrics can be applied in the 1-NN classifier to train the sub-training set. Furthermore, the performance evaluation of the similarity measure can be transformed into a comparison of the classification error rate of the 1-NN classifier under different distance measures. The generalization capacities of the 1-NN classifier with different distance metrics were compared in Figure 11 for the sub-test set by the classification error rate. The bar distribution reflected that the distance based on the improved DTW had lower classification error rates for the two sub-test datasets than that of the other distance measure functions, which also meant that the proposed distance metric was more suitable for similarity evaluation.

## 3. Similarity Measurement for On-Site Vibration Monitoring

### 3.1. Vibration Sequences Acquisition and Preparation

**Figure 12:**
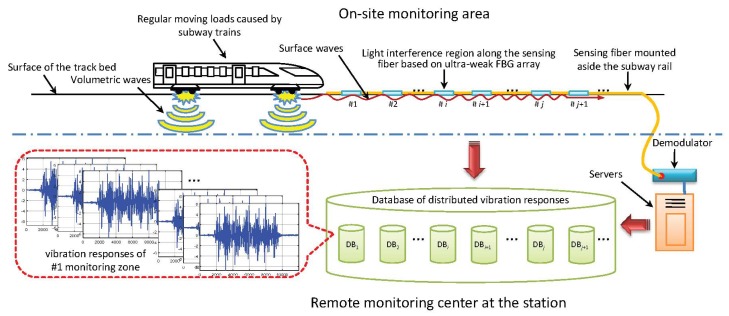
The processing sketch from ultra-weak fiber Bragg grating (FBG) array to distributed vibration.

**Figure 13:**
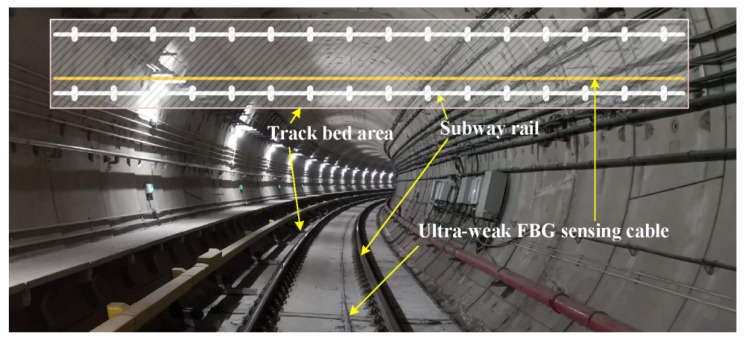
Field layout of ultra-weak FBG sensing cable used for detecting distributed vibration.

As shown in Figure 12, the regular moving loads caused by subway trains can be regarded as a vibration source. Owing to such excitation, the surface waves propagate omni-directionally on the ground. Because the surface wave couples to the track bed and subway rail, a distributed sensing optic fiber mounted beside the rail along the on-site monitoring area can detect the vibration and can be used to establish the vibration database for each monitoring zone. Figure 13 showed part of the actual engineering scenario in the subway tunnel. The monitoring area of interest covered three underground stations with a total length of nearly three kilometers. According to the spatial resolution of the sensing optic fiber and the on-spot layout of the tunnel structure, more than 500 vibration regions along the track bed can be distinguished based on the interrogated address of the light interference [54]. Here, the repeatability of the demodulator was revealed in [55] and the layout of the monitoring system can refer to [15]. When a train passed, the real-time vibration response triggered in each monitoring zone was fully transmitted back to the platform monitoring center at a sampling rate of 1 kHz and processed by the demodulator and servers. Therefore, the database of vibration response caused by passing train can be established for each monitoring zone and the location code of the monitoring area can be used as a unique label of each vibration sequence database.

**Figure 14:**
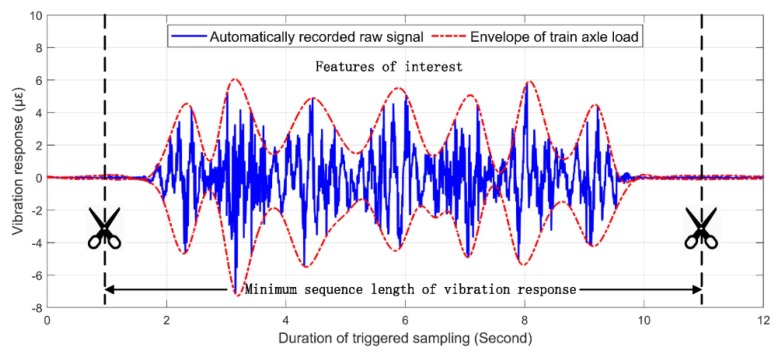
Typical vibration response of a monitoring zone caused by a passing train.

Figure 14 demonstrates the typical vibration responses of a track bed area due to a passing subway train. The triggered vibration responses of each monitoring area automatically recorded due to the passing of the train were basically within 12 s [15]. The characteristics of the vibration response were mainly composed of pulses with a duration of about 9 s caused by the axle weight. To meet the requirement that the node number of the input samples in the SDAE network must be consistent, the main vibration characteristics caused by the action of the train axle in each sample were retained. The sampling points at both ends of the vibration response were then truncated to match the minimum sequence length of the vibration response. Finally, min-max normalization [56] was used to normalize all the vibration amplitudes to the range of 0~1, which can boost a better learning efficiency for the SDAE network.

### 3.2. Result Analysis and Discussion

**Figure 15:**
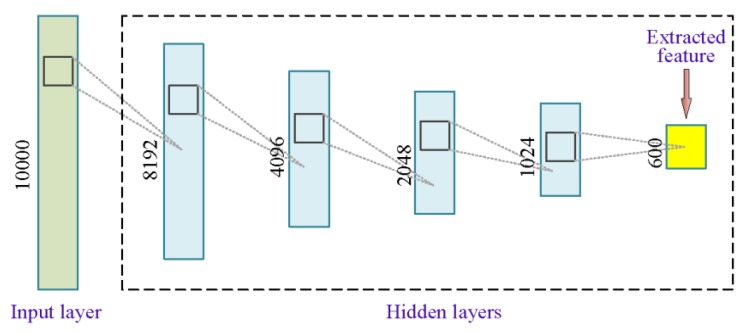
Hidden layers schematic of the SDAE network used for processing vibration responses.

Considering the processing power of current experimental hardware that was composed of a graphics processing unit (GPU) core (GTX 1080 Ti) with twelve 2.20 GHz processors (Intel Xeon E5-2650 v4), we collected the distributed vibration responses caused by 100 passing trains within 2 h in the subway, aiming at three randomly selected monitoring zones labeled #130, #135 and #145 to perform similarity measurement through training the SDAE network and searching the optimal constraint bandwidth. The three selected zones belong to the common track bed in the same traveling area and the ultra-weak FBG sensors used to detect vibration were installed with the consistent craft [15]. First of all, all samples were truncated into the sequence with 10,000 dimensionalities and processed by the min-max normalization. Based on the step-by-step parameter tuning, the most appropriate SDAE network structure assessed by the silhouette coefficient is shown in Figure 15, which set the denoising coefficient to 0.2, contained 5 hidden layers and reduced the input length from 10,000 to 600. The constraint bandwidth was then set to 13 by the 1-NN classifier training. For each set of candidate hyperparameters, it took approximately 2–2.5 h to perform the task on feature extraction and bandwidth search of the 100 groups of datasets of the three monitoring zones.

After completing the two-step training of feature extraction of the SDAE network and distance measurement of the improved DTW algorithm, the established model can be applied to calculate the similarity of new samples. In another subway operation period, three groups of vibration responses of the #130, #135, and #145 monitoring zones caused by passing trains were collected and used to verify the proposed method of measurement similarity.

**Figure 16:**
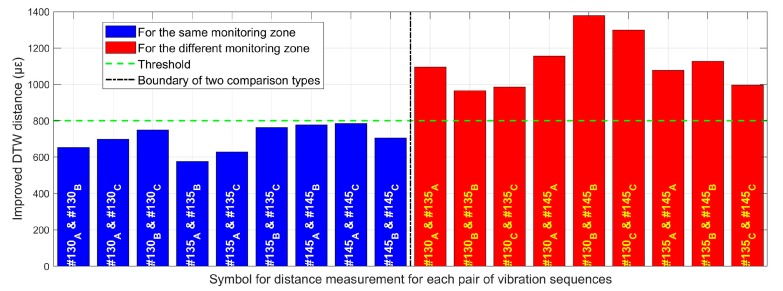
Similarity comparison based on the improved DTW distance.

Figure 16 shows similarity measures between two vibration sequences related to the monitoring zone and the passing train. The left side of the dotted boundary in the bar graph displays the similarities between each pair of vibration responses of the same monitoring zone under different passing trains, while the right side of the boundary shows the similarities of different monitoring areas passed by the same train. Here, the subscripts A, B, and C of the monitoring area numbers in each bar column indicate the different passing trains, and two related monitoring area labels for measuring distance are connected by the symbol ‘&’. Obviously, the threshold of the two comparison types represented by the distance derived from the improved DTW algorithm can be identified, and it was about 800 με. The distance unit here depended on the vibration signal denoted by the strain-induced phase variation between two ultra-weak FBGs [10]. Because the results in the left part of Figure 16 are all below the threshold, it is quantitatively revealed that the similarity of the vibration response at the same physical location in the underground structure is significantly higher than the measurement results between different locations. Moreover, by using the mean distance based on the improved DTW algorithm, the similarities for monitoring zones #130, #135 and #145 can be determined as 700 με, 656 με and 756 με, respectively. The quantitative outcomes based on distance measure not only indicated that the similarity of the structural vibration changed with the location of the underground structure but also revealed that the proposed method can effectively quantify and distinguish such similarity difference. Hence, the condition change of the surrounding structure and environment can be tracked by similarities of structural vibration detected by distributed ultra-weak FBG array.

## 4. Conclusions

This study proposed a similarity measure method to quantify the distributed vibration responses of underground structures, which involved feature extraction by the SDAE network and distance measurement by the improved DTW algorithm. Combining two datasets of one-dimensional time series from UCR archives, the detailed implementation processes for the similarity measure were introduced, and the advantages of feature extraction and distance measure in the proposed method were revealed according to algorithm comparisons. Considering the current processing capabilities of the experimental hardware, the size of the field dataset used to train the SDAE network was limited, but the subsequent experimental outcomes on distance measure still agreed well with the expected cognition. The prediction results of similarity based on the modeling of 100 groups of vibration sequences in three monitoring zones on the subway site demonstrated that the vibration similarity of the same monitoring zone was significantly higher than that from different ones. Moreover, the similarities of distributed vibration closely related to the physical location of the underground structure can be distinguished effectively by the improved DTW distance, demonstrating that the proposed method assisted with the distributed vibration detected by the ultra-weak FBG array is promising for quantifying structural status and locating structural anomalies.

## Figures and Tables

**Table 1:** Experimental datasets for validation method.

Name of Datasets	Sequence Length	Size of the Dataset A	Size of the Dataset B	Number of Labels
CinCECGTorso	1639	1050	350	4
SemgHandMovementCh2	1500	675	225	6

**Table 2:** Datasets for searching the constraint bandwidth and evaluating the distance metrics.

Name of Datasets	Sequence Length	Size of the Sub-Training Set	Size of the Sub-Test Set	Number of Labels
CinCECGTorso	164	50	300	4
SemgHandMovementCh2	150	25	200	6
